# FPGA implementation and voice encryption application of a new hyperchaotic system with high complexity

**DOI:** 10.1038/s41598-025-34605-z

**Published:** 2026-01-07

**Authors:** Khaled Benkouider, Miroslav Mahdal, Sundarapandian Vaidyanathan, Esteban Tlelo-Cuautle, Brisbane Ovilla Martínez, Sezgin Kaçar, Mehmet Ordukaya, Aceng Sambas

**Affiliations:** 1 Laboratory of Automatic and Signals of Annaba (LASA), Badji Mokhtar-Annaba University, P.O. Box 12, Annaba, 23000 Algeria; 2 Department of Control Systems and Instrumentation, Faculty of Mechanical Engineering, VSB-Technical University of Ostrava, 17. Listopadu 2172/15, 70800 Ostrava, Czech Republic; 3 Centre for Control Systems, Vel Tech University, Vel Nagar, Avadi, Chennai, 600062 Tamil Nadu India; 4 Centre of Excellence for Research, Value Innovation and Entrepreneurship (CERVIE), UCSI University, UCSI Heights, Cheras, 56000 Kuala Lumpur Malaysia; 5 Department of Electronics, INAOE, Tonantzintla, 72840 Puebla, Mexico; 6 Department of Computer Science, Center for Research and Advanced Studies, Av. IPN 2508, 07360 Mexico City, CDMX Mexico; 7 Sakarya University of Applied Sciences, Technology Faculty, Electrical and Electronics Engineering, Sakarya, Turkey; 8 Sakarya University of Applied Sciences, Graduate Education Institute, Electrical and Electronics Engineering, Sakarya, Turkey; 9 Faculty of Informatics and Computing, Universiti Sultan Zainal Abidin, Gong Badak, 21300 Terengganu Malaysia; 10 Department of Mechanical Engineering, Universitas Muhammadiyah Tasikmalaya, Tasikmalaya, 46196 Jawa Barat Indonesia

**Keywords:** Engineering, Mathematics and computing

## Abstract

This research work describes a new 4-D hyperchaotic system with high complexity. The proposed 4-D system is hyperchaotic as it has a bounded attractor set with two positive Lyapunov exponents. The large positive Lyapunov exponents of the new 4-D hyperchaotic system exhibit the high complexity of the proposed system. We also show that two nearly identical trajectories quickly separate, with significant differences emerging within just 0.6 seconds, which illustrates the high sensitivity of the state trajectories of the proposed hyperchaotic systems to initial conditions and significant unpredictability. Multistability for the newly proposed hyperchaotic system is illustrated by plotting two different coexisting hyperchaotic attractors for the identical values of the system’s parameters, but for non-identical initial conditions. We also explore offset boosting, a control strategy that allows us to convert the bipolar signal into a unipolar signal without changing the dynamics, which makes the proposed system more adaptable for a range of applications. The electronic implementation of the new system with high complexity is done herein by applying the well-known Euler’s method, which is implemented on a field-programmable gate array (FPGA). In this 4-D system, the nonlinear terms are the multiplication of two state variables that appear in each ordinary differential equation. The FPGA design for the newly developed hyperchaotic system with high complexity is implemented on the Zybo Z7-20 development board with xc7z020clg400-1. In this study, the potential of the new 4D hyperchaotic system in the field of information security was investigated and encryption of voice data was performed using the newly developed hyperchaotic system with high complexity. The variables of the system were evaluated with differential entropy analysis and high randomness levels were verified. The XOR-based encryption algorithm developed using these variables obtained with numerical solutions provides effective protection at time, frequency and statistical levels and allows lossless recovery of data.

## Introduction

In chaos theory, a hyperchaotic system is defined as a dynamical system with a bounded attractor set on which there are at least two positive Lyapunov exponents^1^. The complex nature of chaotic and hyperchaotic systems makes them valuable tools for cryptographic applications, particularly in generating secure keys and reinforcing encryption methods^2,3^. Chaotic and hyperchaotic systems have been widely employed to enhance the protection of diverse forms of information with encryption, such as image encryption^4,5^, voice encryption^6,7^ and video encryption^8,9^. When incorporated into encryption frameworks, chaos increases algorithmic complexity, thereby making unauthorized decryption significantly more challenging^10,11^. The use of chaotic and hyperchaotic systems is not limited to secure communication. Chaotic and hyperchaotic dynamical systems have also been adopted in a wide range of applications such as lasers^12^, memristors^13^, neural networks^14^, machine learning^15^, chemical reactors^16^, circuits^17^, etc.

Several types of chaotic systems with unique characteristics have been modelled in the chaos literature such as hyperjerk systems^18,19^, chameleon systems^20,21^, chaotic systems with unstable nodes^22^, megastable multiscroll attractors^23^, low-power chaotic oscillators^24^, chaotic waveform generators^25^ and multi-scroll 2D chaotic oscillators^26^. In 2021, Joshi et al.^18^ proposed an *n*-th-order simple hyperjerk system with an unstable equilibrium and utilized it as a random pulse generator (RPG), thereby extending hyperjerk system applications into engineering practice. In 2025, Choubey et al.^24^ proposed a design methodology for a low-power, low-voltage, inductor-less Chua’s chaotic oscillator, demonstrating further advancements in compact and efficient chaos-based circuits. In 2025, Gupta et al.^25^ developed a compact, high-frequency memristor emulator circuit capable of both wave shaping and signal generation, demonstrating strong potential for chaos-based hardware applications. In 2025, Ding et al.^26^ introduced a chaotic system capable of producing 2-D grid multi-scroll chaotic attractors through a quasi-sine function, offering new possibilities in multi-directional attractor generation.

In the recent years, significant interest has been shown in the chaos literature in the modelling of hyperchaotic dynamical systems with high complexity^27–39^. In 2020, Prakash et al.^27^ proposed a 4-D hyperchaotic system with a saddle-point index-2 equilibrium and discussed FPGA based applications. In 2020, Trikha and Jahanzaib (2020)^28^ dealt with a 4-D hyperchaotic system with a non-hyperbolic equilibrium and presented its applications in secure communication systems. In 2022, Al-Azzawi and Al-Hayali^29^ discussed the coexistence of attractors and multistability in a new 4D hyperchaotic Sprott-S system consisting of a single equilibrium. In 2023, Vaidyanathan et al.^30^ presented synchronization results associated with a new double-scroll 4-D hyperchaotic system with a saddle-point equilibrium. In 2023, Liu et al.^31^ derived a new 4-D hyperchaotic biplane system and discussed Hopf bifurcation and stability properties of their hyperchaotic system. In 2023, Fu et al.^32^ described the applications of a new 4-D hyperchaotic system in audio encryption. In 2023, Cui and Li^33^ derived a 4-D hyperchaotic four-wing system. In 2024, Liu et al.^34^ designed an image encryption algorithm based on a new 4-D hyperchaotic dynamical system. In 2024, Shukur et al.^35^ presented new encryption results based on a new 4-D hyperchaotic dynamical system with two exponential nonlinearities. In 2024, Chen et al.^36^ presented FPGA design of a new memristor-based hyperchaotic system. In 2024, Al-Azzawi and Hasan^37^ discussed multistability and hybrid projective synchronization results for a new 4-D hyperchaotic system which was developed from a 3-D Lorenz-like chaotic system. In 2025, Borah et al.^38^ presented synchronization results for a new 4-D hyperchaotic financial system. In 2025, Iqbal and Wang^39^ conducted an analysis of a novel fractional-order hyperchaotic system, focusing on its dynamics, stability and synchronization properties.

In this research work, we derive a new 4-D hyperchaotic system by modifying the chaotic dynamics of the 3-D Qi system^40^. We show that the new 4-D hyperchaotic system exhibits the Lyapunov exponents (LEs) $L_1 = 28.168$, $L_2 = 12.453$, $L_3 = 0$ and $L_4 = -176.519$. The proposed 4-D system is hyperchaotic as it has a bounded attractor set with two positive LEs.. The large positive LEs of the new hyperchaotic system exhibit the high complexity of the proposed system. We also show that two nearly identical trajectories quickly separate, with significant differences emerging within just 0.6 seconds, which illustrates the high sensitivity of the state trajectories of the proposed hyperchaotic system to initial conditions and significant unpredictability. The high complexity of the proposed hyperchaotic system enables potential applications of the hyperchaotic system in areas such as secure communications^41,42^, encryption^43,44^, cryptosystems^45,46^, pseudo-random number generation^47–49^, etc.

For a chaotic system, Lyapunov exponents (LEs) are specified in order to quantify the rate at which the nearby state trajectories in state space of a dynamical system converge or diverge. Especially, the existence of a positive Lyapunov exponent (LE) specifies sensitive dependence on initial conditions of the dynamical system, which is a characteristic of a chaotic system. A hyperchaotic system is equipped with the presence of two or more positive Lyapunov exponents (LEs), which pinpoint a higher level of complexity than a chaotic system with a single value of a positive Lyapunov exponent. The Kaplan-Yorke dimension $D_K$ is determined from the Lyapunov exponents (LEs) of a dynamical system.

A 3-D dissipative chaotic system is characterized by the existence of a positive Lyapunov exponent $L_1$, a zero Lyapunov exponent ($L_2 = 0$) and a negative Lyapunov exponent $L_3$ with $L_1 + L_2 + L_3 < 0$. For this chaotic system, the Kaplan-Yorke dimension $D_K$ is defined as

1 $$\begin{aligned} D_K = 2 + \frac{1}{| L_3 |} \, (L_1 + L_2) \end{aligned}$$

A 4-D dissipative hyperchaotic system is characterized by the existence of two positive Lyapunov exponents $L_1 \ge L_2$, a zero Lyapunov exponent ($L_3 = 0$) and a negative Lyapunov exponent $L_4$ with $L_1 + L_2 + L_3 + L_4 < 0$. For this hyperchaotic system, the Kaplan-Yorke dimension $D_K$ is defined as

2 $$\begin{aligned} D_K = 3 + \frac{1}{| L_4 |}\, (L_1 + L_2 + L_3) \end{aligned}$$

In general, a higher value of $D_K$ for a chaotic or a hyperchaotic system pinpoints a more intricate and space-filling attractor, which specifies a greater degree of complexity in the dynamics of the underlying dynamical system^50,51^. A hyperchaotic system with a large value of Kaplan-Yorke dimension indicates that the hyperchaotic attractor occupies a larger portion of the state space, which pinpoint a more complex shape of the attractor^50,51^.

For the proposed hyperchaotic system, the value of $D_K$ is found as $D_K = 3.2301$, which gives a quantitative measure of the complexity of the hyperchaotic attractor, based on its Lyapunov exponents.

In this research work, it is also shown that the proposed hyperchaotic system is equipped with properties such as multistability and offset boosting control. For chaotic or hyperchaotic systems, multistability stands for the coexistence of multiple attractors for the underlying systems when the initial conditions are changed without changing the system parameters^52,53^. Multistability for the proposed hyperchaotic system is illustrated by plotting two different coexisting hyperchaotic attractors for the same values of the system’s parameters, but for two different initial conditions. Offset boosting is an important matter for the control of chaotic systems due to its broadband property and polarity control^54,55^. In this work, we also illustrate new results for the offset boosting for the proposed hyperchaotic system.

The electronic implementation of a chaotic system like the new 4-D hyperchaotic system with high complexity requires the discretization of the ordinary differential equations (ODEs), which can be performed by applying Euler’s numerical method or classical fourth-order Runge-Kutta method. One can also find the discretization of other 4-D, 5-D and 6-D hyperchaotic systems by applying numerical methods for the FPGA implementation, as shown in^1,56–58^. In addition, the FPGA implementation has also been performed for 5-D systems^59,60^, 6-D system^61^, and other higher order systems. As one can infer, the usefulness of the FPGAs is not only for fast verification of a chaotic system but also for the development of chaos-based applications, such as image encryption^1,56^, cancer modeling^58^, and cryptanalysis^61^.

The deterministic nature of chaotic systems and their sensitivity to initial conditions make them attractive for modern cryptography applications. In particular, hyperchaotic systems have superior security potential compared to traditional chaotic systems by offering higher levels of complexity and larger key spaces due to multiple positive Lyapunov exponents. In recent years, various studies have been conducted on the effectiveness of these systems in voice data encryption. Alanazi et al.^62^ developed a speech encryption method based on the G-T chaotic system, effectively hiding the structural features of the signal and providing high security. Similarly, Adhikari and Karforma^63^ successfully encrypted audio data with a chaotic key sequence formed by combining Henon and Tent maps. Babu et al.^64^ performed audio data encryption using the synchronization of fractional order hyperchaotic systems. Farsana et al.^65^ presented a study on audio data encryption using the Fast Walsh Hadamard Transform and mixed chaotic key streams. Mokhnache et al.^66^ implemented speech encryption with a combined chaotic system inspired by classical logistic and cubic maps. These studies show that chaotic systems provide practical and secure solutions for voice data encryption. In addition, the histogram-based differential entropy analysis proposed by Beirlant et al.^67^ is an effective tool to quantitatively evaluate the amount of information and randomness contained in these systems. Within the framework of information theory, data structures with high entropy are more resistant to attacks. In this context, the proposed 4D hyperchaotic system, built on the entropy approaches in the existing literature, offers an effective solution for secure encryption of voice data.

Chaos-based voice encryption has recently advanced along two complementary lines: key-stream generation from chaotic dynamics for lightweight XOR pipelines and PRNG-driven schemes that emphasize randomness quality. Prior studies span 3-D variable-order fractional neural networks with key-stream + XOR and a metric set including Entropy, Correlation, RMS, PSNR, RSS, and MSE^68^, 3-D four-wing chaotic systems that feed a PRNG for lightweight XOR and report Entropy, Correlation, PSNR, MSE, MAXERR, and L2RAT^69^ and 4-D fractional-order hyperchaotic memristor oscillators using key-stream + XOR evaluated mainly by Correlation, Entropy, RMS, and RSS^70^. This research work employs a 4-D hyperchaotic system with a key-stream + XOR block-sample processing pipeline and reports comprehensive metrics (Entropy, Correlation, RMS, PSNR, MSE, MAXERR, and L2RAT) to jointly assess randomness under a unified protocol. This positioning keeps the dimensional complexity of the recent work by Jahanshahi et al.^70^, while broadening the evaluation beyond the recent works^68–70^, enabling clear comparisons across chaos types and encryption flows. Table 1 includes a basic literature comparison for chaotic voice encryption.

**Table 1:** Comparison of recent chaotic voice encryption studies.

Study	Chaotic system type	Dimension	Key encryption method	Main evaluation metrics
Wang et al.^68^	Variable-order fractional Neural network	3-D	Key-stream + XOR / block-sample processing	Entropy, Correlation, RMS, PSNR, RSS, MSE
Benkouider et al.^69^	Four-wing chaotic system	3-D	PRNG + lightweight XOR voice encryption	Entropy, Correlation, PSNR, MSE, MAXERR, L2RAT
Jahanshahi et al.^70^	Fractional-order hyperchaotic memristor system	4-D	Key-stream + XOR / block-sample processing	Correlation, Entropy, RMS, RSS
** This work**	**Hyperchaotic System**	**4-D**	**Key-stream + XOR / block-sample processing**	**Entropy, Correlation, RMS, PSNR, MSE, MAXERR, L2RAT**

## A new 4-D hyperchaotic system with high complexity

In 2005, Qi et al.^40^ proposed a 3-D chaotic system with the dynamics

3 $$\begin{aligned} \left\{ \begin{array}{ccl} \dot{x} & = & a (y - x) + y z \\ \dot{y} & = & c x - y - x z \\ \dot{z} & = & x y - b z \\ \end{array} \right. \end{aligned}$$

Qi et al.^40^ showed that the 3-D system (3) is *chaotic* for the values of the parameters $a = 35$, $b = \frac{8}{3}$ and $c = 80$. For the initial condition $x(0) = 0.1,$ $y(0) = 0.1$, $z(0) = 0.1$, and the parameters $(a, b, c) = \left( 5, \frac{8}{3}, 80 \right)$, the LEs of the Qi system (3) were calculated for $T = 1E5$ seconds as

4 $$\begin{aligned} L_1 = 4.0655, \ L_2 = 0, \ L_3 = -42.7318 \end{aligned}$$

Moreover, the Kaplan-Yorke dimension of the Qi system (3) can be evaluated as

5 $$\begin{aligned} D_K = 2 + \frac{1}{| L_3 |} \, (L_1 + L_2) = 2.0951, \end{aligned}$$

which gives an indicator of the complexity of the Qi chaotic system (3) .

In this research work, a new four-dimensional hyperchaotic system is proposed by introducing additional nonlinear terms and extending the Qi chaotic system (3) with a new dynamic state variable *w*. Specifically, the first equation of the Qi system (3) is modified by enhancing the nonlinearity through a scaling of the cross-product term and by incorporating a linear feedback from the new variable *w*. Moreover, the differential equation for *z* is restructured with a coefficient *d* and an additive coupling with the state *y*, thereby enriching the system’s complexity. Finally, the fourth differential equation $\dot{w} = d (yz + x) + z$ introduces further nonlinear interaction among the existing state variables, enabling the emergence of hyperchaotic behavior.

Thus, we propose the new 4-D dynamics as follows:

6 $$\begin{aligned} \left\{ \begin{array}{ccl} \dot{x} & = & a (2 y - 2 x + y z) - b w \\ \dot{y} & = & c x - y - x z \\ \dot{z} & = & d (x y - z) + y \\ \dot{w} & = & d (y z + x) + z \\ \end{array} \right. \end{aligned}$$

In (6), $X = (x, y, z, w)$ is the 4D state vector, and *a*, *b*, *c*, *d* are bifurcation parameters introduced as tuning constants within the mathematical model. We note that *a*, *b*, *c*, *d* are mathematical constants only and they do not have any physical interpretation. The values of the parameters *a*, *b*, *c*, *d* are carefully selected to generate and control the hyperchaotic behavior of the proposed system (6).

In this paper, we show that the system (6) exhibits a hyperchaotic attractor for the values

7 $$\begin{aligned} a = 50, \ b = 65, \ c = 70, \ d = 35 \end{aligned}$$

For the initial state $X(0) = (0.1, 0.1, 0.1, 0.1)$ and $(a, b, c, d) = (50, 65, 70, 35)$, the 4-D system (6) has the following values of Lyapunov exponents (LEs), which were calculated in MATLAB using $T = 1E5$ seconds:

8 $$\begin{aligned} L_1 = 28.168, \ \ L_2 = 12.453, \ \ L_3 = 0, \ \ L_4 = -176.519 \end{aligned}$$

From the LE values given in (8), it is clear that the proposed system (6) is strongly hyperchaotic with large LE values given by $L_1 = 28.168$ and $L_2 = 12.453$. Moreover, the Kaplan-Yorke dimension of the proposed system (6) is computed as follows:

9 $$\begin{aligned} D_K = 3 + \frac{1}{| L_4 |} \, (L_1 + L_2 + L_3) = 3.2301 \end{aligned}$$

The large value of $D_K$ shows the high complexity of the 4-D hyperchaotic system (6).

Clearly, the 4-D hyperchaotic system (6) has an equilibrium point at the origin, $X_0 = (0, 0, 0, 0)$.

For the hyperchaotic case $(a, b, c, d) = (50, 65, 70, 35)$, the Jacobian matrix of the proposed system (6) at $X_0$ is found as

10 $$\begin{aligned} J_0 = \left[ \begin{array}{cccc} -100 & 100 & 0 & -65 \\ 70 & -1 & 0 & 0 \\ 0 & 1 & -35 & 0 \\ 35 & 0 & 1 & 0 \\ \end{array} \right] \end{aligned}$$

which has the spectral values $-35.0186$, $-135.3041$, 0.5256 and 33.7970. This shows that $X_0$ is a saddle-point equilibrium and unstable. Hence, the hyperchaotic system (6) exhibits self-excited attractors.

**Figure 1:**
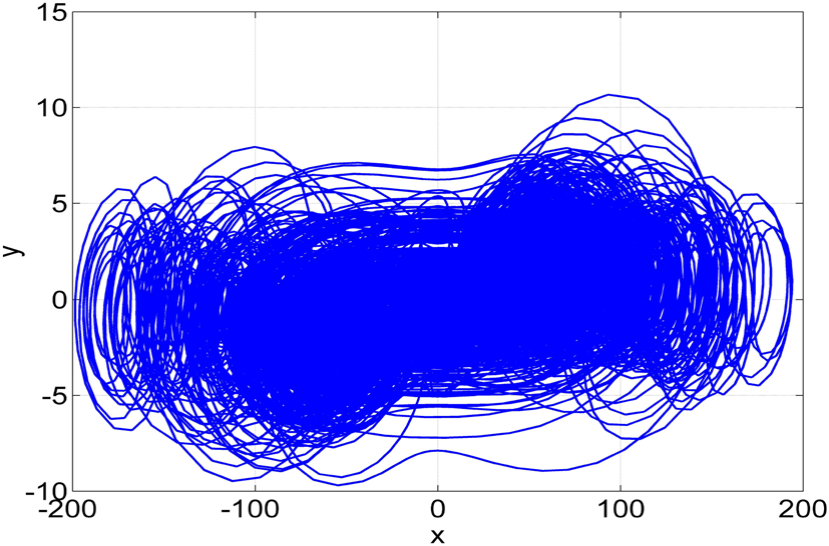
Phase plot in (*x*, *y*) plane of the hyperchaotic attractor of the 4-D system (6) for $(a, b, c, d) = (50, 65, 70, 35)$ and $X(0) = (0.1, 0.1, 0.1, 0.1)$.

**Figure 2:**
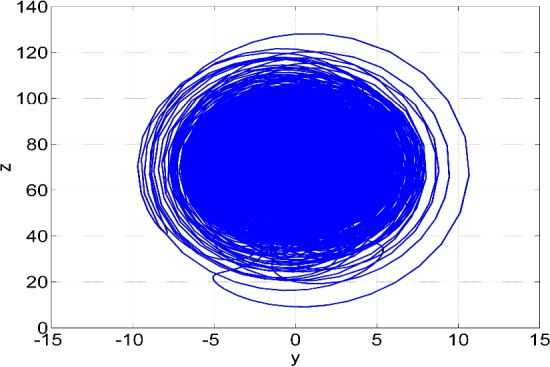
Phase plot in (*y*, *z*) plane of the two-scroll attractor of the system (6) for $(a, b, c, d) = (50, 65, 70, 35)$ and $X(0) = (0.1, 0.1, 0.1, 0.1)$.

**Figure 3:**
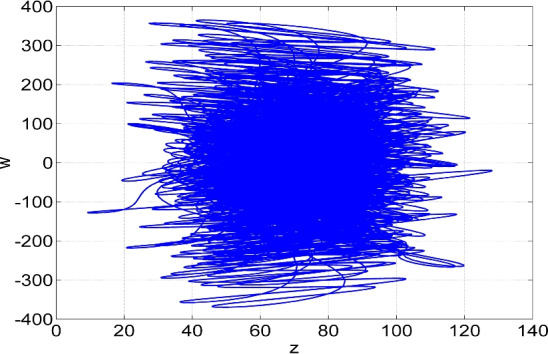
Phase plot in (*z*, *w*) plane of the two-scroll attractor of the system (6) for $(a, b, c, d) = (50, 65, 70, 35)$ and $X(0) = (0.1, 0.1, 0.1, 0.1)$.

**Figure 4:**
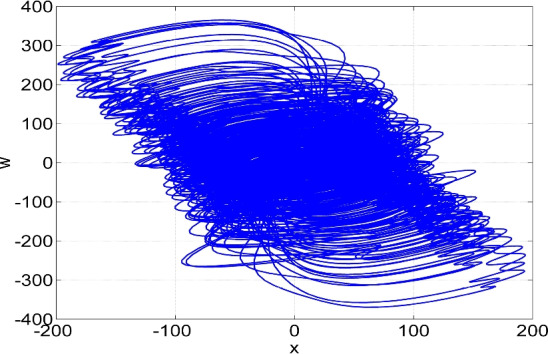
Phase plot in (*x*, *w*) plane of the two-scroll attractor of the system (6) for $(a, b, c, d) = (50, 65, 70, 35)$ and $X(0) = (0.1, 0.1, 0.1, 0.1)$.

Figures 1, 2, 3 and 4 show the MATLAB signal plots of the new 4-D hyperchaotic system (6) with high complexity for the parameter values taken as $(a, b, c, d) = (50, 65, 70, 35)$ and $X(0) = (0.1, 0.1, 0.1, 0.1)$.

## Comparative analysis for the new hyperchaotic system

Chaotic systems are a fascinating class of dynamical systems that exhibit highly complex behavior characterized by extreme sensitivity to initial conditions. In these systems, even a tiny difference in the initial states of two trajectories can lead to dramatically divergent outcomes as time progresses. This phenomenon, commonly known as the “butterfly effect,” emphasizes the unpredictability and non-repeatability of chaotic systems. Hyperchaotic systems are chaotic systems with two or more positive Lyapunov exponents (LEs) and they exhibit a high level of complexity.

**Figure 5:**
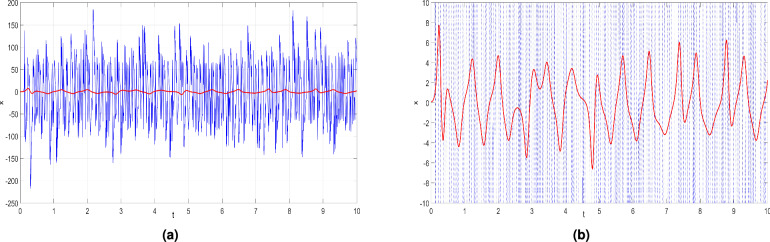
Comparison of time series: new hyperchaotic system (6) (blue) vs. hyperchaotic chen system^71^. (red).

**Figure 6:**
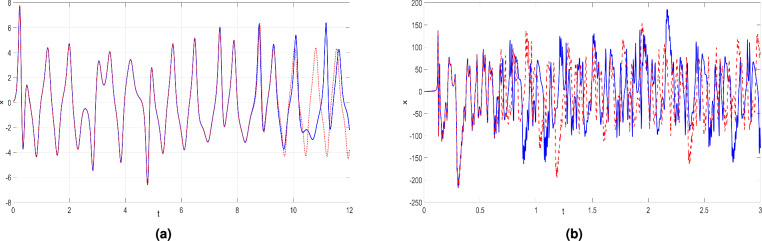
Time response for the state *x* under two very close initial conditions $X_0 = (0.1, 0.1, 0.1, 0.1)$ and $Y_0 = (0.1001, 0.1, 0.1, 0.1)$ of: (**a**) Hyperchaotic Chen system^71^ and (**b**) the new 4-D hyperchaotic system (6).

For the purpose of comparison, we have plotted the time series of the first state variable of the new hyperchaotic system (6) and that of the hyperchaotic Chen system reported in^71^ for the same running time ($t=10s$). Figure 5a showcases the entire time series of both systems, while Fig. 5b provides a zoomed-in view of the time series of the hyperchaotic Chen system^71^. Upon comparing the behavior of the first variable of the new system (6) with that of the hyperchaotic Chen system^71^, a stark contrast becomes evident. The variable of the new system (6) exhibits an exceedingly rapid rate of change, while the hyperchaotic Chen system’s variable changes at a much slower pace. This significant difference in the dynamics of the two systems highlights their distinct characteristics and illustrates the very complex nature of our proposed system (6). Thus, the new system (6) has more complex properties than the hyperchaotic Chen system^71^.

We also performed a comparative analysis by selecting two nearly identical initial conditions: $X_0 = (0.1, 0.1, 0.1, 0.1)$ and $Y_0 = (0.1001, 0.1, 0.1, 0.1)$ . These initial conditions were used for both the proposed hyperchaotic system (6) and the hyperchaotic Chen system^71^. The results are illustrated in Fig. 6, where the solid line corresponds to the *x*-trajectory starting from the initial condition $X_0 = (0.1, 0.1, 0.1, 0.1)$, while the dashed line represents the *x*-trajectory from $Y_0 = (0.1001, 0.1, 0.1, 0.1)$. As shown in Fig. 6a for the hyperchaotic Chen system^71^, the two nearby *x*- trajectories remain relatively close even at $t = 12$seconds. In contrast, in Fig. 6b, the nearby *x*-trajectories of the new hyperchaotic system (6) quickly diverge from each other, becoming separate at $t \approx 0.6$ seconds, and within the time range $t \in [0.6,3]$, they become significantly different. This demonstrates that the new hyperchaotic system (6) is highly sensitive to initial conditions, and predictability is lost quickly.

Furthermore, an important characteristic of our system is that its largest LE can reach 28, and the second positive LE is over 12, both of which are significantly larger than those observed in previously reported hyperchaotic systems. Table 2 presents a comparison of some typical recently reported hyperchaotic systems along with their corresponding LEs. It is evident from the table that the largest LE of the newly proposed hyperchaotic system (6) is greater than the LE of the recent 4-D hyperchaotic systems reported in Table 2. Additionally, the second positive LE of the new system (6) is notably larger than those found in other systems reported in Table 2, where most systems have very small second LE that are less than one. As a result, the trajectories of the new system (6) expand much faster than those of other 4-D systems reported in Table 2, demonstrating significantly stronger hyperchaotic behavior.

**Table 2:** Comparison table: Lyapunov exponents of some recently reported 4-D hyperchaotic systems.

Number	Hyperchaotic system	${L_1}$	${L_2}$	${L_3}$	${L_4}$
1.	Prakash et al. (2020)^27^	2.1726	0.0149	0	$-15.0990$
2.	Trikha and Jahanzaib (2020)^28^	0.4582	0.0144	0	$-1.4713$
3.	Al-Azzawi and Al-Hayali (2022)^29^	0.2041	0.0023	0	$-1.2056$
4.	Vaidyanathan et al. (2023)^30^	3.0717	0.0527	0	$-18.1354$
5.	Liu et al. (2023)^31^	16.9402	3.4133	0	$-3.4133$
6.	Fu et al. (2023)^32^	0.2027	0.1091	0	$-26.2692$
7.	Cui and Li (2023)^33^	4.2770	0.0910	0	$-46.3640$
8.	Liu et al. (2024)^34^	9.0715	1.6860	0	$-38.8661$
9.	Shukur et al. (2024)^35^	0.2371	0.0247	0	$-1.0309$
10.	Chen et al. (2024)^36^	14.1490	8.4210	0	$-63.4750$
11.	Al-Azzawi and Hasan (2024)^37^	3.0743	0.0068	0	$-23.0379$
12.	Borah et al. (2025)^38^	1.6896	1.4902	0	$-30.4801$
13.	Iqbal and Wang (2025)^39^	2.1747	0.0190	0	$-17.3006$
14.	Our proposed system	28.1680	12.4530	0	${-176.5190}$

## Exploring the dynamic behavior of the new hyperchaotic system

In this section, we focus on an in-depth exploration of the dynamic features exhibited by the proposed system (6), accomplished through meticulous numerical computations. The behaviors of the proposed system are scrutinized in relation to its various parameters, employing a comprehensive array of scientific methods, including Lyapunov characteristic exponent spectra, bifurcation diagrams, and phase plots. Our aim is to gain profound insights into the intricate behavior of the system (6) and to understand how it responds to different parameter settings.

### Dynamic behavior analysis with the varied parameter *a*

The behavior of the constructed system (6) will be explored in this section while considering *a* as the control constant. Here, we will demonstrate the influence of varying *a* between 10 and 50 on the dynamics of the system (6) while keeping the remaining constants as $b = 65$, $c = 70$ and $d = 35$.

**Figure 7:**
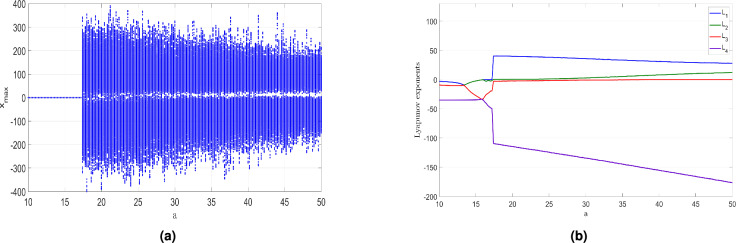
MATLAB plots showing (**a**) bifurcation results and (**b**) LEs of the newly developed system (6) when $a \in [10, 50]$, $b = 65$, $c = 70$ and $d = 35$.

**Figure 8:**
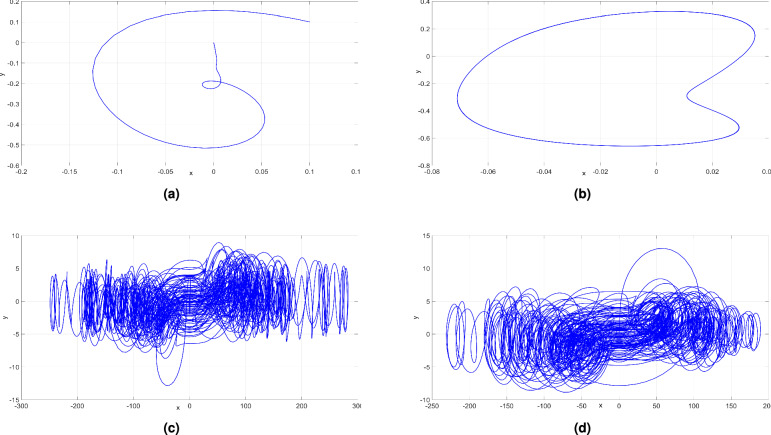
(**a**) (*x*, *y*)-point attractor of (6) when $a = 12$, (**b**) (*x*, *y*)-periodic attractor of (6) when $a = 17$, (**c**) (*x*, *y*)-chaotic attractor of (6) when $a = 18$, and (**d**) (*x*, *y*)-hyperchaotic attractor of (6) when $a = 35.$.

The bifurcation diagram shown in Fig. 7a and the Lyapunov exponents (LEs) shown in Fig. 7b reveal that the newly developed hyperchaotic system (6) demonstrates a wide range of behaviors when *a* is varied. The bifurcation plot and Lyapunov indices shown in Fig. 7 provide compelling evidence supporting this observation. Affected by changes in *a*, the system (6) demonstrates diverse dynamical phenomena, including periodic attractor, chaotic attractor with a high value of the Maximum LE (MLE), hyperchaotic attractor with two large positive LEs, or convergence to a stable state. These findings bring out the remarkable influence of changes in the system (6) with respect to variations in the parameter $a \in [10, 50]$.

When $a \in [10, 16]$, as depicted in Fig. 7b, it is clearly seen that $L_i < 0$ for $i = 1, 2, 3, 4$. This observation confirms that the system (6) tends toward an equilibrium state, thereby displaying stable dynamics within this range of control parameter. Specifically, when $a = 12$, we observe the (*x*, *y*)- attractor of system (6) in Fig. 8a. The associated LEs were found as follows: $L_1 =-4.617$, $L_2=-10.261$, $L_3=-10.265$, and $L_4=-34.856$.

In the range where $a \in [16, 17.2]$, as depicted in Fig. 7b, $L_1 = 0$, while $L_i < 0$ for $i = 1, 2, 3$. This implies that the 4D system (6) exhibits periodic dynamics, a finding further supported by the bifurcation diagram in Fig. 7a. To explore this behavior, we specifically chose the control parameter *a* to be 17. Consequently, we observe the (*x*, *y*) periodic attractor in Fig. 8b. The associated LEs were found as follows: $L_1=0$, $L_2=-2.391$, $L_3=-19.464$, and $L_4=-48.143$.

As *a* varies in the range [17.2, 23], the dynamics of the 4D system (6) undergoes a significant transformation, transitioning from a periodic state to chaos, as evidenced in Fig. 7a. Notably, the MLE assumes a positive and remarkably large value, suggestive of the high complexity of the newly developed 4-D system (6) in its chaotic behavior. Additionally, we get the Kaplan-Yorke dimension of the newly developed system (6) as $D_K = 3.3449$, a clear demonstration of the generation of intricate chaotic behavior within this particular range of *a*. Specifically, when $a = 18$, the (*x*, *y*) attractor of the system (6) is depicted in Fig. 8c, clearly showcasing the chaotic behavior exhibited by the system (6). The associated LEs for this chaotic attractor were found as follows: $L_1=40.720$, $L_2=0$, $L_3=-2.590$, and $L_4=-110.558.$

In the range where *a* varies in [23, 50], the newly developed system (6) undergoes a transition from chaos to hyperchaos behavior, featuring two large positive LEs. This implies that the system’s dynamics exhibit rapid expansions in two independent directions within the phase space leading to highly intricate hyperchaotic behavior. Specifically, when $a = 35$, the hyperchaotic attractor of the newly developed system (6) in (*x*, *y*) plane is visualized in Fig. 8d and represented by a fractional value of the Kaplan-Yorke dimension $D_K=3.2703.$ The associated LEs were found as follows: $L_1=33.661$, $L_2=5.456$, $L_3=0$, and $L_4=-144.703$.

Table 3 provides a detailed summary of the influence of the parameter *a* on the properties of the newly developed system (6).

### Dynamic behavior analysis with the varied parameter *b*

The behavior of the constructed system (6) will be explored in this section while considering *b* as the control constant. Here, we will demonstrate the influence of varying *b* between $-3$ and 65 on the dynamics of the system (6) while keeping the remaining constants as $a = 50$, $c = 70$ and $d = 35$.

**Figure 9:**
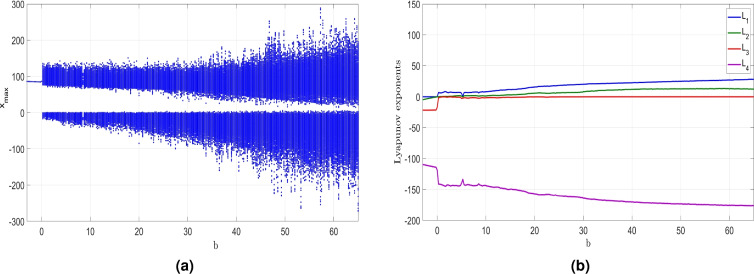
MATLAB plots showing (**a**) Bifurcation results and (**b**) LEs of the newly developed system (6) when $b \in [-3, 65$, $a = 50$, $c = 70$ and $d = 35$.

**Figure 10:**
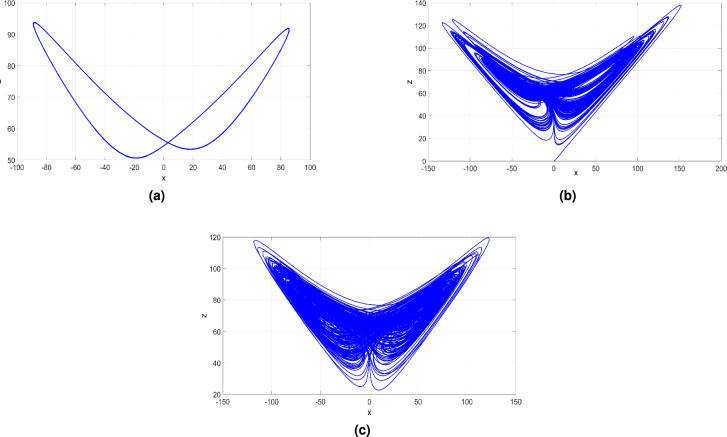
(**a**) (*x*, *z*)-periodic orbit of (6) when $b = 2$, (**b**) (*x*, *z*) chaotic attractor of (6) when $b = 0.4$ and (**c**) (*x*, *z*) hyperchaotic attractor of (6) when $b = 20$.

The bifurcation diagram shown in Fig. 9a and the Lyapunov exponents (LEs) shown in Fig. 9b reveal that the newly developed hyperchaotic system (6) demonstrates a wide range of behaviors when *b* is varied. The bifurcation plot and Lyapunov indices shown in Fig. 9 provide compelling evidence supporting this observation.

When the parameter *b* takes values from $-3$ to 0, the newly developed system (6) presents periodic behavior as evidenced by a zero MLE and validated by the bifurcation diagram shown in Fig. 9. Specifically selecting $b = -2$ allows us to visualize the (*x*, *z*)- periodic attractor in Fig. 10a and determine the corresponding LEs as $L_1=0$, $L_2=-2.922$, $L_3=-21.804,$ and $L_4=-111.320$.

Within the interval [0, 5.2], the dynamical features of the newly developed system (6) undergo a transition from periodicity to chaos, with a large positive MLE indicating high complexity. The (*x*, *z*) attractor in Fig. 10b is plotted for $b = 0.4$, highlighting the chaotic behavior. The Lyapunov exponents provide valuable insights into the dynamic nature of the 4-D system (6) in this parameter range, obtained as follows: $L_1=6.550$, $L_2=0$, $L_3=-0.989$, and $L_4=-141.661$. Additionally, the Kaplan-Yorke dimension has a fractional value of $D_K = 3.0393$.

In the range from 5.3 to 65, the dynamical features of the newly developed system (6) experience a shift from chaos to hyperchaos behavior, defined by the existence of two large positive LEs signifying a high level of complexity. The (*x*, *z*) highly hyperchaotic attractor of the 4-D system (6) depicted in Fig. 10c corresponds to a *b* value of 20. Specifically, when $b = 20$, the LE values for the newly developed system (6) were obtained as follows: $L_1=15.744$, $L_2=6.920$, $L_3=0$, and $L_4=-157.448$. Additionally, we find that $D_K = 3.1439$, further emphasizing the complexity of the system’s behavior in this region.

Table 3 provides a detailed summary of the influence of the parameter *b* on the properties of the newly developed system (6).

### Dynamic behavior analysis with the varied parameter *c*

**Figure 11:**
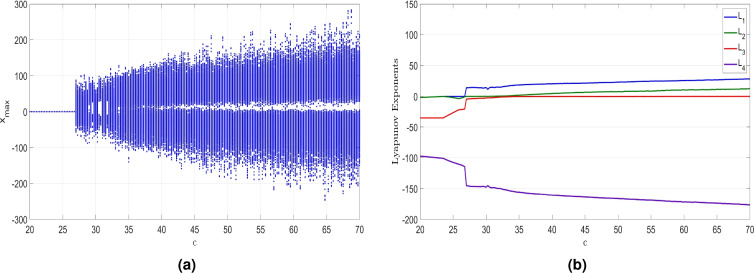
MATLAB plots showing (**a**) Bifurcation results and (**b**) LEs of the newly developed system (6) when $c \in [20, 70]$, $a = 50$, $b = 65$ and $d = 35$.

**Figure 12:**
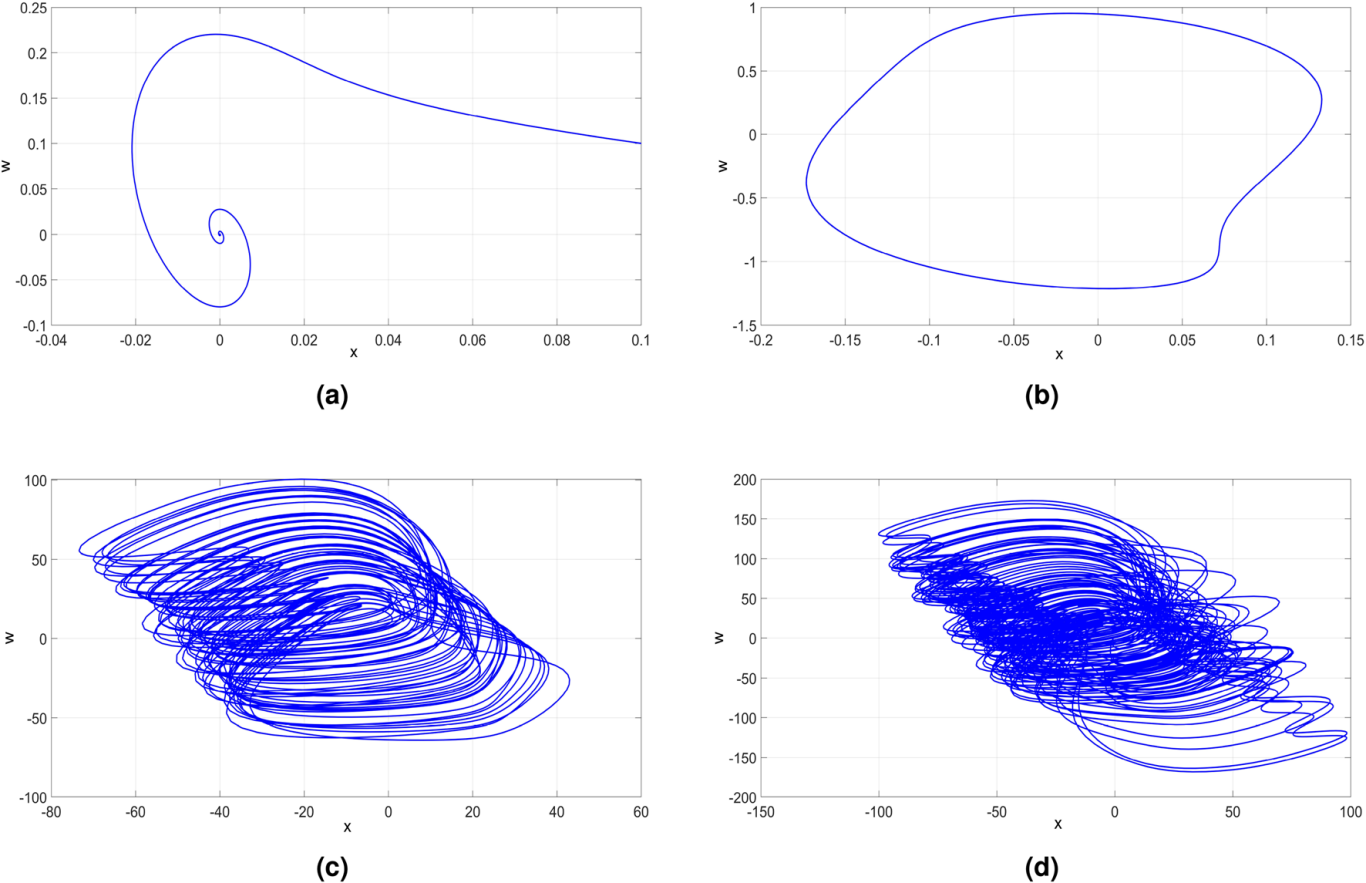
(**a**) (*x*, *w*)-point attractor of (6) when $c = 20.5$, (**b**) (*x*, *w*)-periodic attractor of (6) when $c = 25.5$, (**c**) (*x*, *w*)-chaotic attractor of (6) when $c = 28$, and (**d**) (*x*, *w*)-hyperchaotic attractor of (6) when $c = 35$.

The bifurcation diagram shown in Fig. 11a and the Lyapunov exponents (LEs) shown in Fig. 11b reveal that the newly developed hyperchaotic system (6) demonstrates a wide range of behaviors when *c* is varied. Here, we will demonstrate the influence of varying *c* between 20 and 70 on the dynamics of the newly developed system (6) while keeping the remaining constants as $a = 50$, $b = 65$ and $d = 35$. Within this range, the system can either converge to a stable state or exhibit periodic behavior, chaos, and hyperchaos.

Within the interval of *c* values ranging from 20 to 23.5, as depicted in Fig. 11b, the MLE is negative. This observation indicates the system’s tendency to converge to an equilibrium state, reflecting stable dynamics within this range of *c*. Particularly, when $c = 20.5$, Fig. 12a showcases the attractor of the newly developed system (6) in the (*x*, *w*) plane. The associated LEs were found as follows: $L_1=-1.533$, $L_2=-1.534$, $L_3=-35.018$, and $L_4=-97.840$.

Within the range of *c* values between 23.5 and 26.75, the MLE of the system (6) is zero, while the other LEs are negative. This observation implies that system (6) exhibits periodic behavior, a finding further supported by the bifurcation analysis plot presented in Fig. 11a. Specifically, we select $c = 25.5$ to explore this periodic behavior further. As a result, we observe the periodic attractor in the (*x*, *w*) plane as shown in Fig. 12b. The associated LEs for this attractor were found as follows: $L_1=0$, $L_2=-2.930$, $L_3=-23.696$, and $L_4=-109.197$.

In the range from 26.75 to 32, the dynamical features of the newly developed system (6) undergo a shift from periodicity to chaos, with a large MLE indicating high complexity. The (*x*, *w*) phase plot of the system (6) for $c = 28$ is shown in Fig. 12c, clearly illustrating the chaotic behavior. The associated LEs for (6) were obtained as follows: $L_1=14.381$, $L_2=0$, $L_3=3.526$, and $L_4=-146.679$. Additionally, we find that $D_K = 3.0714$, further confirming the chaotic nature of the system’s behavior.

In the range from 32 to 70, the system’s dynamical features undergo a shift from chaotic to hyperchaotic behavior, marked by the existence of two large LEs implying high complexity. The hyperchaotic attractor is simulated in (*x*, *w*) plane and depicted in Fig. 12d corresponds to the *c* value of 35. The corresponding LEs of (6) were obtained as follows: $L_1=18.457$, $L_2=1.886$, $L_3=0$, and $L_4=-155.870$. Additionally, we find that $D_K = 3.1305.$

Table 3 provides a detailed summary of the influence of the parameter *c* on the properties of the newly developed system (6).

### Dynamic behavior analysis with the varied parameter *d*

**Figure 13:**
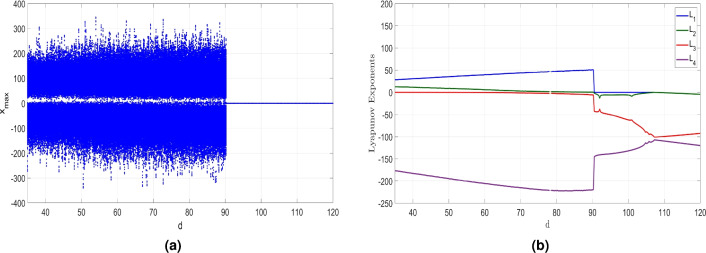
MATLAB plots showing (**a**) Bifurcation results and (**b**) LEs of the newly developed system (6) when $d \in [35, 120]$, $a = 50$, $b = 65$ and $c= 70$.

**Figure 14:**
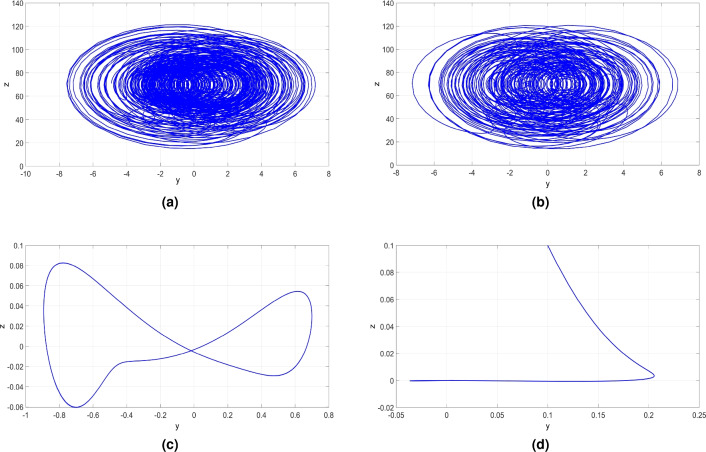
(**a**) (*y*, *z*) hyperchaotic attractor of (6) when $d = 60$, (**b**) (*y*, *z*) chaotic attractor of (6) when $d = 90$, (**c**) (*y*, *z*)-periodic attractor of (6) when $d = 95$, and (**d**) (*y*, *z*)-point attractor of (6) when $d = 120$.

The bifurcation diagram shown in Fig. 13a and the Lyapunov exponents (LEs) shown in Fig. 13b reveal that the newly developed hyperchaotic system (6) demonstrates a wide range of behaviors when *d* is varied. Here, we will demonstrate the influence of varying *d* between 35 and 120 on the dynamics of the newly developed system (6) while keeping the remaining constants as $a = 50$, $b = 65$ and $c = 70$.

Figure 13 depicts that system (6) can exhibit convergence to a stable state, display periodic behavior, depict chaotic dynamics, or even manifest hyperchaotic behavior.

Within the range of *d* values from 35 to 80, the newly developed system (6) demonstrates hyperchaos, characterized by the presence of two significantly large positive LEs as shown in Fig. 13b, which highlight the system’s highly complex behavior. This complex dynamics is exemplified by the hyperchaotic attractor in the (*y*, *z*) plane for $d = 60$ as depicted in Fig. 14a. Additionally, we find that $D_K = 3.2201$. The associated LEs for this hyperchaotic attractor were obtained as follows: $L_1=39.541$, $L_2=5.712$, $L_3=0$, and $L_4=-205.643$.

Within the range of *d* values from 80 to 90.2, the dynamics of the 4-D system (6) undergoes a shift from hyperchaos to chaos, characterized by the presence of one positive LE as shown in Fig. 13b. The maximum LE is greater than 50, and $D_K = 3.2053$, indicating the generation of complex chaotic dynamics. Specifically, for $d = 90$, Fig. 14b illustrates the chaotic attractor of the newly developed system (6). The associated LEs for this chaotic attractor were obtained as follows: $L_1=50.859$, $L_2=0$, $L_3=-5.761$, and $L_4=-219.674$.

Within the range of *d* values from 90.2 to 107, the MLE is zero, signifying that the system exhibits periodic pattern with no complexity. Specifically, for $d = 95$, Fig. 14c depicts the (*y*, *z*) periodic attractor, confirming the periodic nature of the system (6) within this parameter range. The associated LEs for this periodic attractor were obtained as follows: $L_1=0$, $L_2=-5.960$, $L_3=-50.172$, and $L_4=-138.292$.

Within the range of *d* values from 107 to 120, as illustrated in Fig. 13b, the MLE has a negative value. This observation signifies that the newly developed system (6) tends to converge to an equilibrium state, indicating stable dynamics within this specific range of the control parameter. Specifically, when $d = 120$, Fig. 14d displays the attractor of the system (6) in (*y*, *z*) plane. The associated LEs were obtained as follows: $L_1=-4.420$, $L_2=-4.420$, $L_3=-92.259$, and $L_4=-119.898$.

Table 3 provides a detailed summary of the influence of the parameter *d* on the properties of the newly developed system (6).

**Table 3:** Dynamics, LEs and $D_{K}$ of the newly developed 4-D System (6) versus its Bifurcation Parameters.

Dynamics	Parameters				Bifurcation parameter	Lyapunov exponents				$D_{K}$	Attractor
	*a*	*b*	*c*	*d*		$LE_1$	$LE_2$	$LE_3$	$LE_4$		
Point	[0, 16]	65	70	35	$a=12$	$-4.167$	$-10.261$	$-10.265$	$-34.856$	0	Fig. 8 (a)
	50	65	[20, 23.5]	35	$c=20.5$	$-1.533$	$-1.534$	$-35.018$	$-97.841$	0	Fig. 12 (a)
	50	65	70	[107, 120]	$d=120$	$-4.420$	$-4.421$	$-92.159$	$-119.898$	0	Fig. 14 (d)
Periodic	[16, 17.2]	65	70	35	$a=17$	0	$-2.391$	$-19.464$	$-48.143$	0	Fig. 8 (b)
	50	$[-3, 0], 5.25$	70	35	$b=2$	0	$-2.922$	$-21.8049$	$-111.320$	0	Fig. 10 (a)
	50	65	[23.5, 26.75]	35	$c=25.5$	0	$-2.930$	$-23.696$	$-109.197$	0	Fig. 12 (b)
	50	65	70	[90.2, 707]	$d=95$	0	$-5.960$	$-50.172$	$-138.292$	0	Fig. 14 (c)
Chaos	[17.2, 23]	65	70	35	$a=18$	40.720	0	$-2.590$	$-110.558$	3.3449	Fig. 8 (c)
	50	[0, 5.2]	70	35	$b=0.4$	6.550	0	$-0.989$	$-141.661$	3.0393	Fig. 10 (b)
	50	65	[26.75, 32]	35	$c=28$	14.381	0	$-3.526$	$-146.679$	3.0740	Fig. 12 (c)
	50	65	70	[80, 90.2]	$d=90$	50.859	0	$-5.761$	$-219.674$	3.2053	Fig. 14 (b)
Hyperchaos	[23, 50]	65	70	35	$a=35$	33.661	5.456	0	$-144.703$	3.2703	Fig. 8 (d)
	50	[5.3, 65]	70	35	$b=20$	15.744	6.920	0	$-157.448$	3.1439	Fig. 10 (c)
	50	65	[32, 70]	35	$c = 35$	18.457	1.886	0	$-155.870$	3.1305	Fig. 12 (d)
	50	65	70	[35, 80]	$d=60$	39.541	5.712	0	$-205.643$	3.2201	Fig. 14 (a)
	50	65	70	35	−	28.168	12.453	0	$-176.519$	3.2301	Fig. 1

## Coexistence of attractors: investigating the impact of initial conditions on system behavior

Multistability or coexisting attractors are fascinating nonlinear phenomena in chaos theory, denoting the concurrent formation of multiple distinct attractors originating from different initial states. Through extensive numerical simulations, we have unveiled the remarkable richness of multiple attractors (periodic, chaotic or hyperchaotic) simultaneously generated by the newly developed system (6). Observing how these attractors interact and coexist in the phase phase of the newly developed system (6) is an important study as it gives rise to complex and intertwined dynamics.

We consider two distinct initial points, *Z*01 and *Z*02, for the new 4-D system (6):

$$Z01: (3, 3, 3, 3) \text{[Displayed } \text{ in } \text{ Blue } \text{ color] }$$

$$Z02: (-3, -3, 3, 3) \text{[Displayed } \text{ in } \text{ Red } \text{ color] }$$

These initial points mark the starting positions from which the system’s dynamics (6) will evolve and unfold. The blue trajectory originates from the initial state *Z*01 with coordinates (3, 3, 3, 3), while the red color trajectory corresponds to the initial state *Z*02 with coordinates $(-3, -3, 3, 3)$. As the system (6) evolves over time, the trajectories diverge and unfold in distinct ways, leading to different behaviors and giving rise to the emergence of two different coexisting attractors.

**Figure 15:**
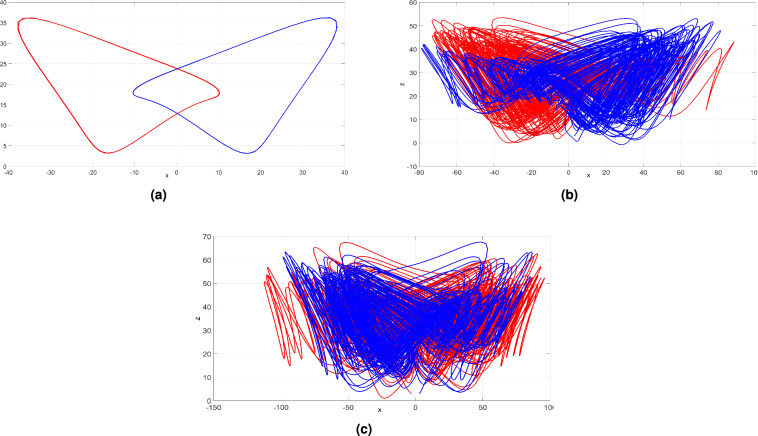
Multiple attractors of the newly developed system (6) in the (*x*, *z*) plane plotted using MATLAB: (**a**) periodic attractors, (**b**) chaotic attractors and (**c**) hyperchaotic attractors.

The newly developed system (6) generates two distinct periodic attractors while keeping the parameters fixed at at $a=50$, $b=65$, $c=21.5$, and $d=35$. Notably, these attractors appear under identical parameter values but originate from different initial conditions as depicted in Fig. 15a.

When $a=50$, $b=65$, $c=28$, and $d=35$, the newly developed system (6) generates two distinct chaotic attractors as depicted in Fig. 15b.

Upon maintaining the parameters at fixed values of $a=50$, $b=65$, $c=35$, and $d=35$, the new system (6) demonstrates the appearance of two distinct hyperchaotic attractors. Interestingly, these hyperchaotic attractors emerge with identical parameter settings but arise from two different initial points, *Z*01 and *Z*02, as depicted in Fig. 15c.

Numerical simulations reveal the coexistence of multiple attractor types, deepening our insight into the multistability features of the newly developed 4-D system (6) and opening up exciting avenues for further research and practical applications.

## Offset boosting behavior

Offset boosting behavior of a dynamical system refers to the ability to adjust the amplitude of the state of a system dynamically through a state feedback mechanism. The offset boosting control (OBC) mechanism does not alter the system’s underlying dynamics; instead, it facilitates the displacement of the attractor from its original position to a new location, either in a positive or negative direction based on the control parameter’s value.

The newly developed 4-D system (6) exhibits a variable-boosting hyperchaotic behavior, as the fourth variable *w* solely appears in the first equation. The variable *w* becomes controllable and is able to be amplified by substituting it with $w + k$. By doing so, the newly developed system (6) can be expressed in the following modified form to incorporate OBC:

11 $$\begin{aligned} \left\{ \begin{array}{ccl} \dot{x} & = & a (2 y - 2 x + y z) - b (w+k) \\ \dot{y} & = & c x - y - x z \\ \dot{z} & = & d (x y - z) + y \\ \dot{w} & = & d (y z + x) + z \\ \end{array} \right. \end{aligned}$$

**Figure 16:**
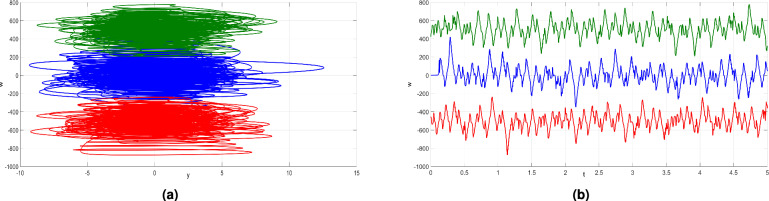
(**a**) Hyperchaotic attractors in the (*y*, *w*)-plane under different *k* values. (**b**) The *w* signal under different *k* values [$k = 0$ (blue), $k = 500$ (red) and $k = -500$ (green)].

The parameter *k* represents an offset boosting controller. When specific values are assigned to the parameters $a=50$, $b=65$, $c=70$, and $d=35$, boosted hyperchaotic attractors can be achieved. In Fig. 16a, we identify different placements of the attractors of (11) in the (*y*, *w*)-planes for different values of *k*.

Notably, when $k < 0$, the attractors of (11) shift in the positive direction, whereas a positive value of *k* causes them to shift in the negative direction. This impacts in a significant transformation of the hyperchaotic signal *w*. This transformation alters the signal from a bipoloar hyperchaotic state to a unipolar hyperchaotic state, as shown in Fig. 16b. This distinctive feature plays a crucial role in confidential data transmission and diverse engineering fields, enhancing the utility and versality of the system (11).

## FPGA implementation of the new 4-D hyperchaotic system

The FPGA implementation of a hyperchaotic system requires to apply a numerical method to discretize the continuous-time ODEs. Is for this reason that the most used method is the well-known Euler’s numerical one. The application of Euler’s method helps to discretize ODEs by applying the iterative equation of the form: $x_{n+1}=x_n+hf(x_n)$, where *h* is the time step. In this manner, the new 4-D strong hyperchaotic system defined in (6) can be discretized by applying Euler’s method to get the iterative equations given in (12). As one can see, those equations perform arithmetic operations associated to multiplication, addition and subtraction, which can be designed in embedded systems such as on an FPGA board. The newly developed 4-D system (6) generates hyperchaotic behavior by setting $a = 50$, $b = 65$, $c = 70$, and $d=35$, with the initial phase vector ($x_0,\ y_0,\ z_0,\ w_0$)=(0.1, 0.1, 0.1, 0.1), and the value of step-size equal to $h= 0.000005$.

12 $$\begin{aligned} \left\{ \begin{array}{ll} x_{n+1}=& x_{n}+h(a(2y_{n}-2x_{n}+y_{n}z_{n})-bw_{n})\\ y_{n+1}=& y_{n}+h(cx_{n}-y_{n}-x_{n}z_{n})\\ z_{n+1}=& z_{n}+h(d(x_{n}y_{n}-z_{n})+y_{n})\\ w_{n+1}=& w_{n}+h(d(y_{n}z_{n}+x_{n})+z_{n}) \end{array} \right. \end{aligned}$$

**Figure 17:**
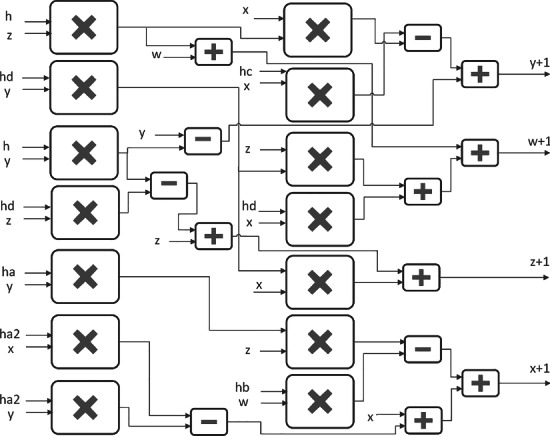
Block description of the new $4-D$ hyperchaotic system (6).

From the discretized equations (12), one can describe the design using blocks, as shown in Fig. 17. In the block description, the inputs are associated to the starting values of the *x*, *y*, *z*, and *w* phase variables, while the outputs are associated to the same state variables after they are evaluated to proceed to the next iteration, so that they are labeled as $x+1$, $y+1$, $z+1$, $w+1$, which correspond to $x_{n+1}$, $y_{n+1}$, $z_{n+1}$, $w_{n+1}$ given in (12). These output values are obtained every two clock cycles and are fed back to the system as the new input to be considered as $x_{n}$, $y_{n}$, $z_{n}$, $w_{n}$. In the block description, *ha*, *hb*, *hc*, and *hd* are associated to the pre-computation of the multiplication of the system parameters by the selected *h*. In the same way, *ha*2 pre-computes the multiplication by two of the parameters *a* by *h*, which is used to calculate $x_{n+1}$.

The FPGA implementation of hyperchaotic dynamical systems is very useful to validate the generation of hyperchaotic attractors, and the digital synthesis can be summarized by listing the hardware resources and the number of clock cycles required to perform all the evaluations, which is associated to the latency. In this manner, using Euler’s method and from (12), the new 4-D hyperchaotic system (6) has been implemented on the Zybo Z7-20 development board with xc7z020clg400-1. Table 4 summarizes the hardware resources that are used from the block description given in Fig. 17.

**Table 4:** Hardware resources for the FPGA design of (12) using Xilinx Zybo Z7-20 (xc7z020clg400-1).

Resources	Used	Util
LUTs	3409	6.41%
FFs	2081	1.96%
DSPs	88	21.45%
Max Freq	114 MHz	–

The computer arithmetic has been performed using 64-bit data-path in fixed-point format (10.54). As sketched in Fig. 17, the main blocks are related to multipliers, which are drawn in vertical lines to appreciate the operations, so that the addition and subtraction blocks are responsible for performing the operations of the independent terms resulting from the multiplications. The operations are completed in two clock cycles, thus giving a maximum frequency of operation of 114 MHz.

**Figure 18:**
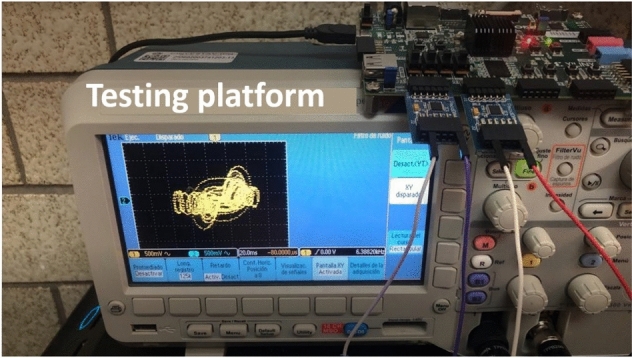
Experimental setup for the FPGA design of the new hyperchaotic system (6).

**Figure 19:**
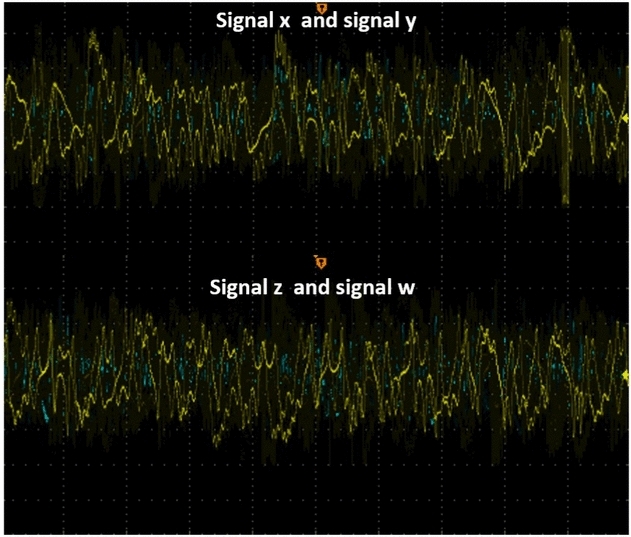
Experimental views for the signal *x*, *y*, *z*, and *w*. The top image shows *x* in yellow and *y* in blue. The bottom image shows *z* in yellow and *w* in blue.

**Figure 20:**
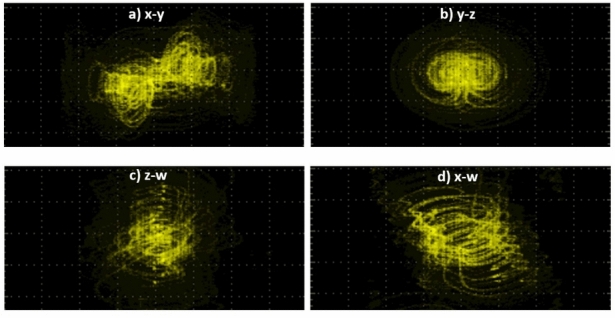
Experimental views for the planes $x-y$, $y-z$, $z-w$, and $x-w$, with $a = 50$, $b = 65$, $c = 70$, and $d=35$. Also, ($x_0,\ y_0,\ z_0,\ w_0$)=(0.1, 0.1, 0.1, 0.1), and $h=0.000005.$.

The experimental setup is shown in Fig. 18, consisting of the FPGA and a digital-to-analog converter from which it is possible to observe the outputs of the new system through the oscilloscope. In this manner, the experimental views of the time series of the four state variables are shown in Fig. 19. Finally, the experimental phase-portraits between the state variables $x-y$, $y-z$, $z-w$, and $x-w$, are shown in Fig. 20.

## Voice encryption application

### Entropy-based randomness analysis

In this study, a differential entropy analysis was performed to determine the amount of information and the degree of randomness contained in the four-dimensional state variables of the system (6). A histogram-based entropy estimation method was applied to the variables containing continuous data. For each variable, a histogram was constructed using 50,000 data points.

Differential entropy was calculated using the following approximate formula ^67^:

13 $$\begin{aligned} \hat{H}(X) \approx - \sum _{i=1}^{n} P_i \log _2(P_i) \cdot \Delta x \end{aligned}$$

where $P_i$ is the normalized probability of the *i*-th 1000, and $\Delta x$ is 1000. The resulting entropy values are presented in Table 5:

**Table 5:** Estimated entropy values of state variables.

Variable	Entropy (bit)
*w*	348.61
*x*	349.13
*y*	350.78
*z*	347.28

According to the obtained results, the entropy for the *x* variable was determined as 349.13 bits, for *y* as 350.78 bits, for *z* as 347.28 bits and for *w* as 348.61 bits. These values are both quite high in terms of absolute magnitude and quite close to each other.

At this point, it is important to emphasize what the entropy values mean in the theoretical context. Entropy is a basic measure that shows the extent to which a system is random and unpredictable in terms of information theory. The data type used in this study consists of 64 bit double-precision floating-point numbers. For such data, the maximum theoretical entropy value that can be achieved under ideal conditions is approximately 64 bits, because this requires each bit to be zero or one with equal probability. However, the calculations made here are differential entropy measurements, unlike the classical Shannon entropy, and for continuously distributed data, this value can reach much higher values depending on the unit of measurement. Therefore, the fact that the entropy values are in the range of 347–351 bits indicates that the data produced by the system has a very complex, wide-ranging, and highly random distribution.

In addition, the fact that the four variables have entropy values that are quite close to each other indicates that the information distribution of the system in the multidimensional space is homogeneous, that is, each dimension contributes to the encryption process at a similar rate. This is a highly desirable feature for multidimensional key generation or chaotic system-based encryption algorithms^72,73^. High entropy increases the level of masking structural information in both time and frequency domains, especially in the encryption of analog data such as audio, and strengthens the resistance against possible cryptanalytic attacks^67^. In this respect, the entropy performance of the proposed hyperchaotic system reveals a strong cryptographic potential not only at the numerical level but also at the theoretical level.

Thus, the proposed hyperchaotic system (6) is very suitable for cryptographic applications.

### Encryption and decryption processes

**Figure 21:**
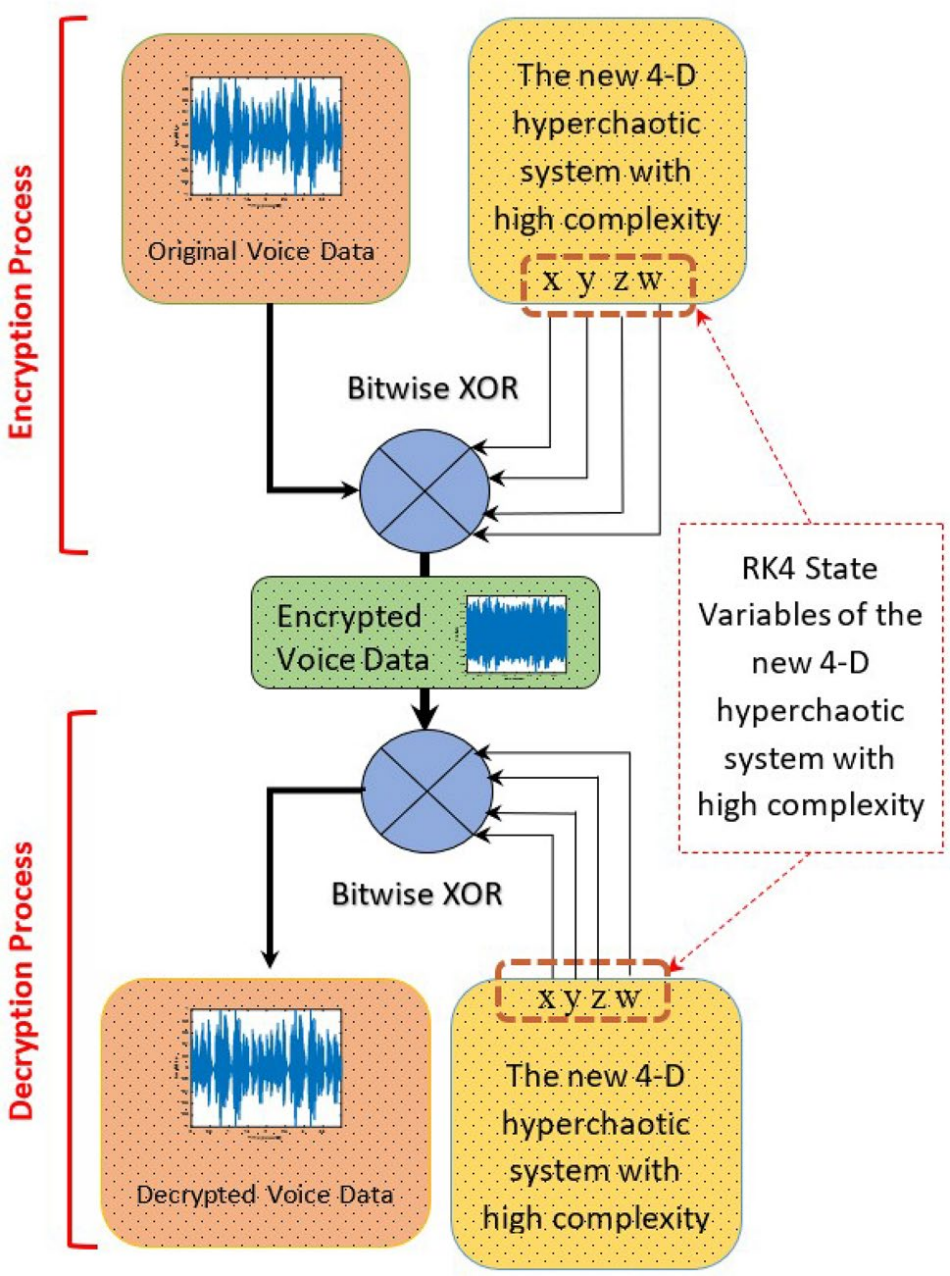
Encryption-decryption process: block diagram.

In this study, an application was developed for encrypting and decrypting audio data using the newly developed hyperchaotic system (6) that exhibits high-order dynamic complexity. Figure 21 presents the overall structure representing this process.

The encryption process begins with the numerical solution of the hyper-chaotic system’s state variables (*x*, *y*, *z*, *w*) using the fourth-order Runge-Kutta (RK4) method. These variables are generated based on the system’s initial conditions and parameters, and each is represented in 64-bit double-precision floating-point format. Similarly, the original voice data is processed in this format.

A direct bit-wise XOR operation is performed between the 64-bit float numbers generated by the hyper-chaotic system and the 64-bit float numerical representations of the audio data. The resulting new float values constitute the encrypted audio data.

In the decryption process, the same hyper-chaotic system’s state variables are recomputed using the RK4 method. Another bit-wise XOR operation is then applied between the encrypted audio data and the regenerated variables, successfully recovering the original audio data.

This method enables a robust encryption/decryption structure using only numerical and bit-level operations without requiring any format transformation of the data. The proposed approach provides an effective and practical solution for the secure transmission and storage of audio data.

### Experimental results

**Figure 22:**
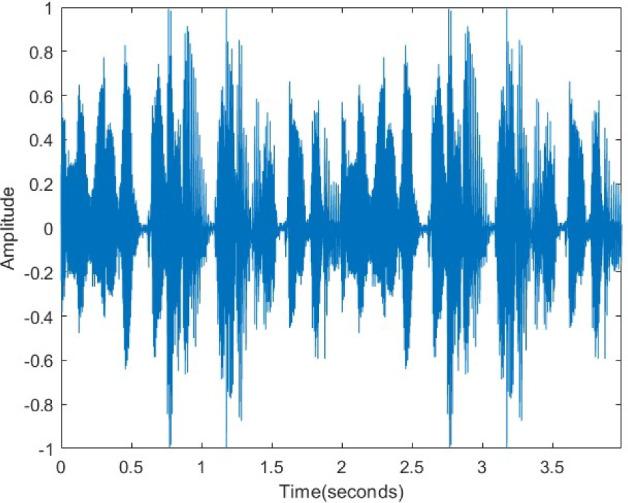
Original voice signal.

**Figure 23:**
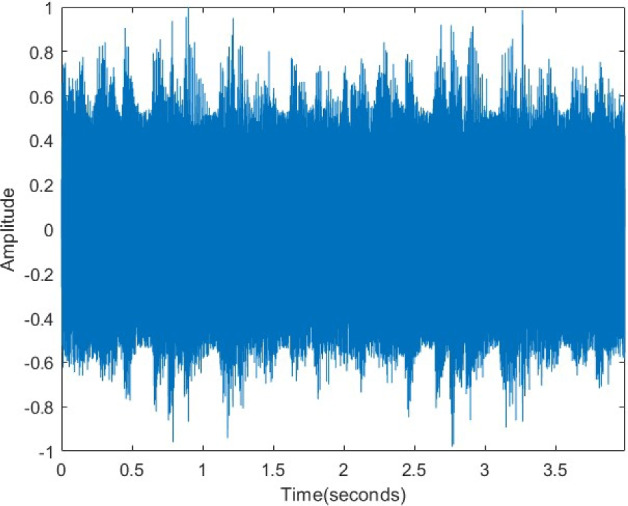
Encrypted voice signal.

**Figure 24:**
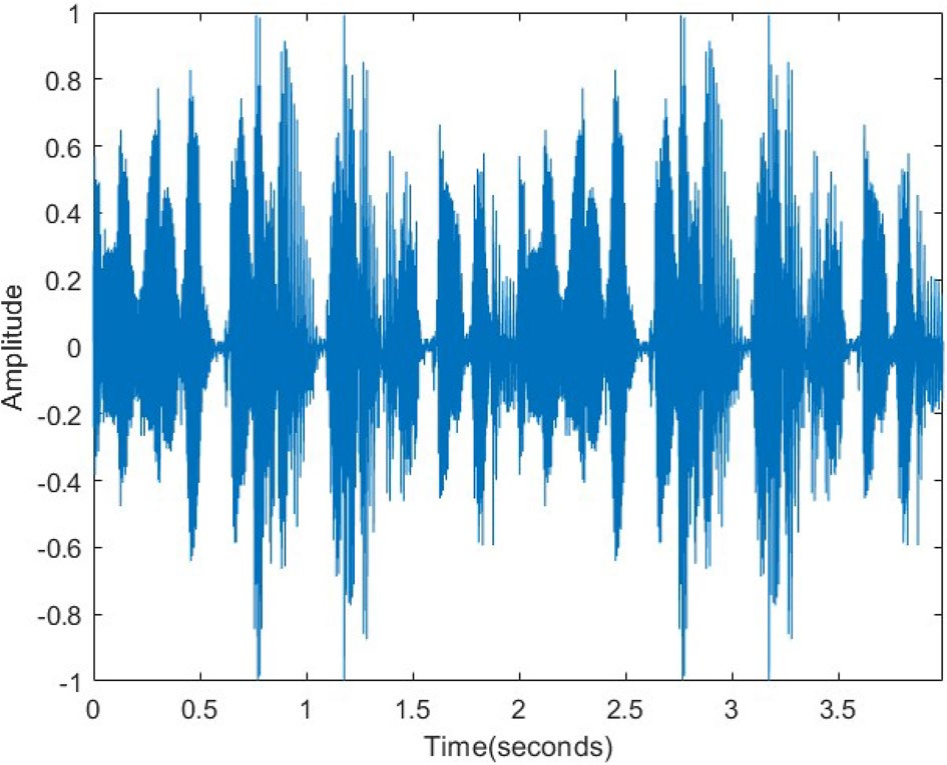
Decrypted voice signal.

In Fig. 22, the original audio signal exhibits a distinct waveform and characteristic amplitude variations. After encryption (Fig. 23), this structure is entirely disrupted, and the signal transforms into a statistically random noise-like appearance. This transformation confirms the effectiveness of the encryption in the time domain. As displayed in Fig. 24, the decrypted signal exactly matches the original waveform, proving that the algorithm preserves data integrity and operates losslessly.

**Figure 25:**
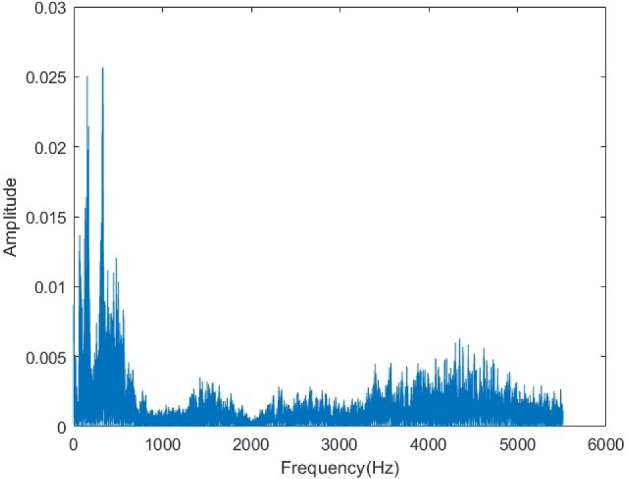
Original voice spectrum.

**Figure 26:**
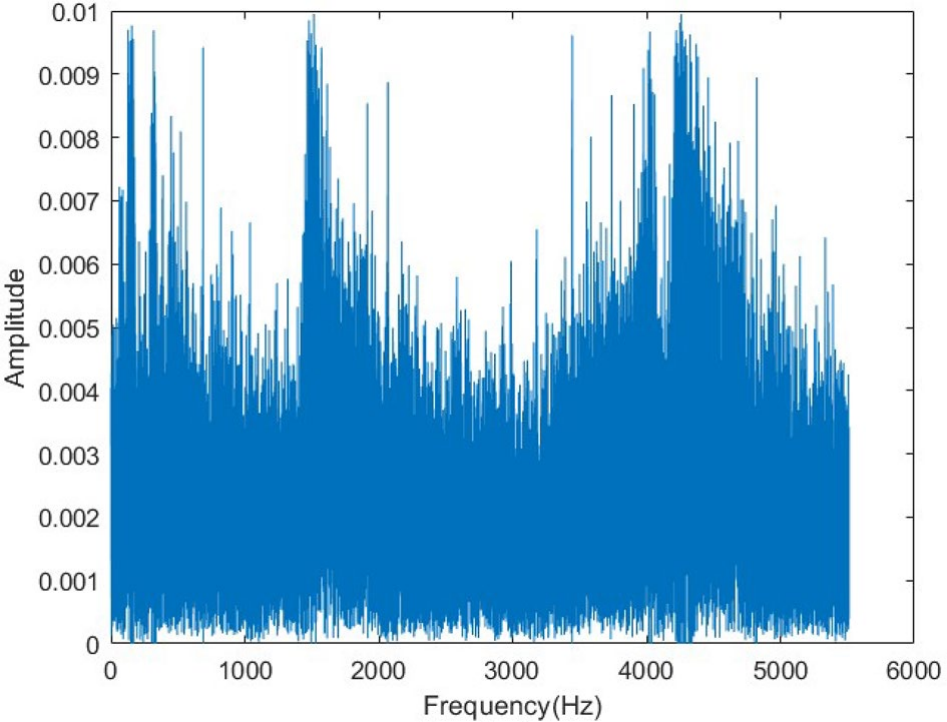
Encrypted voice spectrum.

**Figure 27:**
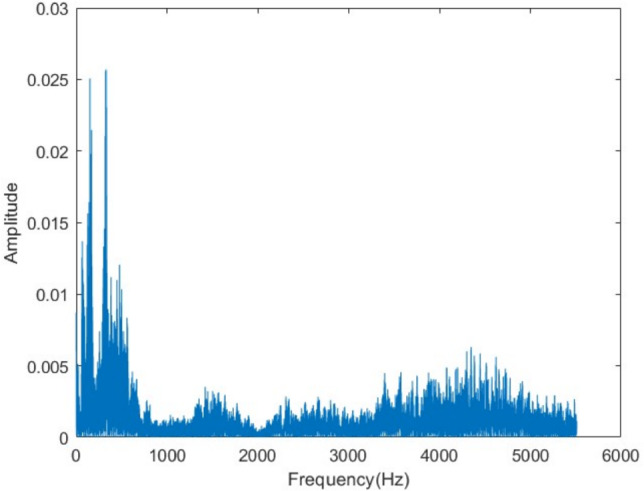
Decrypted voice spectrum.

The original audio spectrum (Fig. 25) displays energy concentration in specific frequency components. In the encrypted spectrum (Fig. 26), these components are completely masked, and energy is distributed uniformly across the entire frequency band. This demonstrates that the encryption effectively conceals the spectral properties of the signal, enhancing its resistance to frequency-domain attacks. The exact restoration of the original spectrum after decryption (Fig. 27) confirms the reliability of the algorithm in the frequency domain.

**Figure 28:**
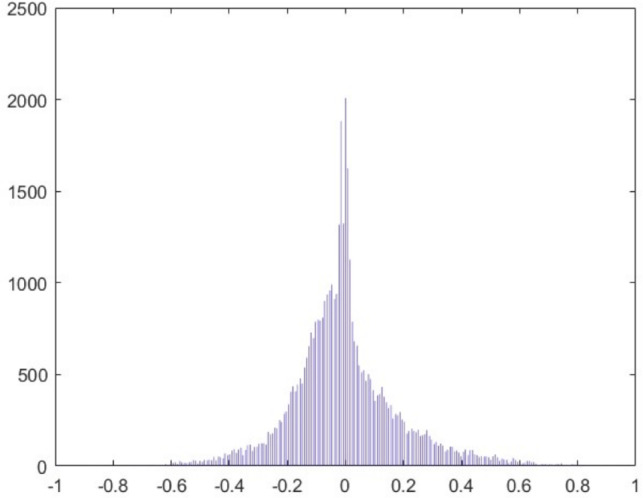
Original voice histogram.

**Figure 29:**
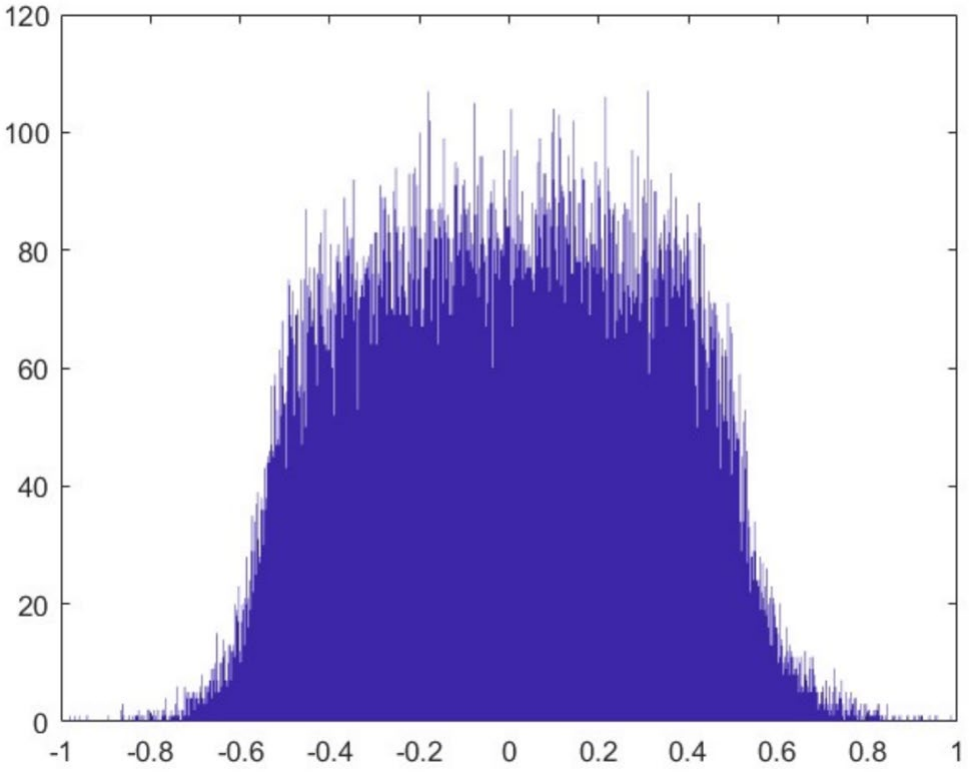
Encrypted voice histogram.

**Figure 30:**
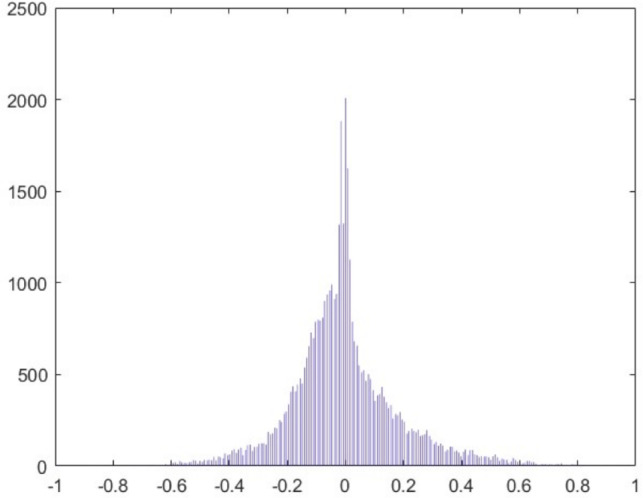
Decrypted voice histogram.

The histogram of the original audio data (Fig. 28) exhibits a distribution concentrated at specific amplitude values. After encryption (Fig. 29), this distribution becomes uniform. This change indicates that the encryption conceals the statistical properties of the data successfully. The histogram of the decrypted data (Fig. 30) fully recovers the original distribution. This result confirms that the algorithm is not susceptible to statistical analysis and preserves data integrity.

The fundamental metrics used in the evaluation of audio encryption algorithms aim to objectively measure the algorithm’s performance in terms of security and data integrity (Table 6). Entropy analysis assesses the randomness level of the encrypted data, thus evaluating its resistance to statistical attacks. High entropy values indicate successful encryption^72^. The correlation coefficient analyzes the linear relationship between the original and encrypted data; values close to zero confirm that structural features have been effectively concealed^72^.

PSNR (Peak Signal-to-Noise Ratio) and MSE (Mean Squared Error) quantitatively express the amount of distortion introduced by the encryption process^72^. The RMS (Root Mean Square) value measures the variations in signal amplitude to evaluate the impact of encryption on dynamic range^74^. MAXERR (Maximum Error) indicates the largest discrepancy between the original and processed signals^74^, while L2RAT quantifies changes in energy distribution^75^. The results obtained using these metrics are presented in Table 6.

**Table 6:** Quantitative performance metrics for voice encryption.

Metric	Original	Encrypted	Decrypted
Entropy	6.4669	14.5203	6.4669
Correlation	0.5034	-0.0223	0.5034
RMS	0.1896	0.3225	0.1896
PSNR	58.0758		
MSE	0.1013		
MAXERR	0.9382		
L2RAT	2.8950		

These metrics enable a comprehensive evaluation of the encryption algorithm. The observed increase in entropy from 6.4669 to 14.5203 after encryption indicates that the data has reached a near-perfect level of statistical randomness. The changes in RMS values (original: 0.1896 $\rightarrow$ encrypted: 0.3225 $\rightarrow$ decrypted: 0.1896) clearly illustrate the algorithm’s effect on the dynamic range. The symmetric pattern of RMS changes suggests that the algorithm maintains a solid balance between security and reversibility. The 70% increase in RMS following encryption aligns with the entropy rise (6.4669 $\rightarrow$ 14.5203), highlighting a consistent transformation of data amplitude.

The drop in correlation coefficient from 0.5034 to -0.0223 confirms that structural relationships were entirely disrupted, while the complete restoration of all metrics after decryption proves that the algorithm operates without data loss. The PSNR value (58.0758 dB) and MSE (0.1013) indicate that the encryption introduces a controlled level of distortion, while the MAXERR (0.9382) value falls within the threshold of imperceptibility to the human eye. The L2RAT value of 2.8950 mathematically confirms the change in energy distribution. Overall, the level of data transformation and recovery performance demonstrated by the algorithm supports its practical applicability.

**Table 7:** Comparison of quantitative performance metrics for chaotic voice encryption.

System	Stage	Entropy	Correlation	RMS	PSNR	MSE	MAXERR	L2RAT
Wang et al.^68^	Original	6.3798	0.3564	0.1786	54.9490	0.2081	–	–
	Encrypted	12.6150	0.4298	0.3848	–	–	–	–
	Decrypted	6.3798	0.3564	0.1786	–	–	–	–
Benkouider et al.^69^	Original	6.4669	0.5034	–	57.0095	0.1295	1.7529	2.6062
	Encrypted	14.4248	0.0101	–	–	–	–	–
	Decrypted	–	–	–	–	–	–	–
Jahanshahi et al.^70^	Original	6.3143	0.5944	0.1776	–	–	–	–
	Encrypted	10.0977	$-0.1670$	0.2751	–	–	–	–
	Decrypted	6.3143	0.5944	0.1776	–	–	–	–
**This Study**	**Original**	**6.4669**	** 0.5034**	**0.1896**	**58.0758**	**0.1013**	**0.9382**	**2.8950**
	** Encrypted**	**14.5203**	$\mathbf {-0.0223}$	**0.3225**	** –**	** –**	**–**	** –**
	**Decrypted**	** 6.4669**	**0.5034**	** 0.1896**	** –**	**–**	** –**	**–**

When the results in Table 7 are compared, the entropy value of the encrypted audio data obtained in this study (14.5203) is higher than all of the recent works^68–70^, indicating that statistical randomness is better achieved after encryption. In terms of correlation coefficient, a value very close to zero was obtained for the encrypted data in this study as -0.0223, which means very low correlation with the original data, while values of 0.4298 were observed in the work by Wang et al.^68^, 0.0101 in the work by Benkouider et al.^69^ and -0.1670 in the work by Jahanshahi et al.^70^. When the RMS values are compared, the RMS after encryption in this study (0.3225) is at an acceptable level between the RMS after encryption values in the recent works^68,70^. In terms of PSNR, the value of this study (58.0758 dB) is higher than the PSNR values in the recent works^68–70^ revealing that the signal quality is better preserved after encryption and decryption. When the MSE values are examined, the error values of 0.1013 is obtained in this study, which is lower than the MSE values in the recent works^68,69^. In the MAXERR and L2RAT metrics, the values of this study (0.9382, 2.8950) exhibit higher accuracy and different error distribution properties compared to the recent work^69^. In general, Table 7 reveals that this study performs better than the recent works^68–70^ in terms of entropy, PSNR and MSE, and the correlation is close to zero, which increases the encryption security.

## Conclusions

In this research work, we proposed a new 4-D hyperchaotic system with the Lyapunov exponents $L_1 = 28.168$, $L_2 = 12.453$, $L_3 = 0$ and $L_4 = -176.519$. The large positive Lyapunov exponents of the new 4-D hyperchaotic system exhibit the high complexity of the proposed system. Furthermore, we also showed that the new 4-D hyperchaotic system exhibits multistability and offset boosting control. The FPGA implementation of the new 4-D hyperchaotic system, has been performed using the FPGA Zybo Z7-20 development board with xc7z020clg400-1. The experimental setup consisted of the FPGA and a digital-to-analog converter to observe the signals in the oscilloscope, which attractors are in good agreement with MATLAB simulations. In view of the high complexity, the proposed 4-D hyperchaotic system has potential applications in secure communications, steganography and cryptosystems. Finally, we designed an voice encryption algorithm with the help of the proposed 4D hyperchaotic system. The performed analyses show that the proposed 4D hyperchaotic system successfully meets the basic security criteria such as high randomness (entropy), low correlation and lossless data return in voice data encryption. Investigations in both time and frequency domains confirm that the algorithm effectively hides the signal structure and is resistant to attacks. These findings reveal that the proposed method provides a secure, effective and practical solution in voice-based data transmission and storage.

## Data Availability

All the data used in this research work are available from the corresponding author on request.
